# Sleep and Cognition in Children with Fetal Alcohol Spectrum Disorders (FASD) and Children with Autism Spectrum Disorders (ASD)

**DOI:** 10.3390/brainsci10110863

**Published:** 2020-11-16

**Authors:** Rabya Mughal, Catherine M. Hill, Anna Joyce, Dagmara Dimitriou

**Affiliations:** 1 Sleep Education and Research Laboratory, Psychology and Human Development, UCL-Institute of Education, London WC1H 0AA, UK; d.dimitriou@ucl.ac.uk; 2 School of Clinical and Experimental Sciences, Faculty of Medicine, University of Southampton, Southampton SO16 6YD, UK; c.m.hill@soton.ac.uk; 3 School of Psychotherapy & Psychology, Regent’s University London, London NW1 4NS, UK; joycea@regents.ac.uk

**Keywords:** sleep, fetal alcohol spectrum disorder, FASD, autism, autism spectrum disorder, ASD, actigraphy, cognition

## Abstract

Children with Fetal Alcohol Spectrum Disorders (FASD) and Autism Spectrum Disorders (ASD) experience significantly higher rates of sleep disturbances than their typically developing peers. However, little is known about the association between sleep and the cognitive phenotype in these clinical populations. Structural damage affecting cortical and subcortical connectivity occurs as a result of prenatal alcohol exposure in children with FASD, whilst it is believed an abundance of short-range connectivity explains the phenotypic manifestations of childhood ASD. These underlying neural structural and connectivity differences manifest as cognitive patterns, with some shared and some unique characteristics between FASD and ASD. This is the first study to examine sleep and its association with cognition in individuals with FASD, and to compare sleep in individuals with FASD and ASD. We assessed children aged 6–12 years with a diagnosis of FASD (*n* = 29), ASD (*n* = 21), and Typically Developing (TD) children (*n* = 46) using actigraphy (CamNTech Actiwatch 8), digit span tests of working memory (Weschler Intelligence Scale), tests of nonverbal mental age (MA; Ravens Standard Progressive Matrices), receptive vocabulary (British Picture Vocabulary Scale), and a choice reaction time (CRT) task. Children with FASD and ASD presented with significantly shorter total sleep duration, lower sleep efficiency, and more nocturnal wakings than their TD peers. Sleep was significantly associated with scores on the cognitive tests in all three groups. Our findings support the growing body of work asserting that sleep is significant to cognitive functioning in these neurodevelopmental conditions; however, more research is needed to determine cause and effect.

## 1. Introduction

Sleep is not simply a cessation of the waking state; rather, it is an active brain state subserving neural health. Accumulating data demonstrate how sleep in childhood contributes to neurodevelopment through its role in neuroplasticity and brain maturation [1]. Sleep-dependent neuronal processes occurring at the cellular level during Rapid Eye Movement (REM) and Non-Rapid Eye Movement (NREM) underlie a number of local and global organisational tasks, including memory consolidation, task learning, and the formation of neural pathways organising visual, auditory, and integrated or abstract events [1,2,3,4,5]. Associations between cognitive processes and sleep deprivation are reported, with chronic sleep disturbances associated with aberrant neural pathways [3,6]. Early intervention is an increasingly important area of therapeutic concern, especially given that sleep interventions can ameliorate behavioural and cognitive outcomes in children [7]. 

FASD is the neurodevelopmental and physical consequence of prenatal alcohol exposure, manifesting in a pattern of significant impairment across three or more domains of brain function (generally ≤2 standard deviations). Domains include executive function, memory, cognition, social/adaptive skills, academic achievement, language, motor, attention, and activity level [8]. Syndromic physical features may also be present [8]. Prenatal alcohol exposure is associated with the inhibited growth, structure, and function of prefrontal and parietal areas resulting in a phenotypic behavioural and cognitive profile [9]. Children with FASD may display attentional problems around vigilance, reaction time, and the speed or inhibition of information processing [10]. In fact, around 60% of children with FASD also fall into the diagnostic category of ADHD, both of which are characterised by patterns of inattentiveness, hyperactivity, and impulsivity [11]. Diminished working memory capacity is intrinsic to FASD and connected to significant deficits in prefrontal, posterior, and parietal lobe connectivity [12]. Sleep disturbances are prevalent in children with FASD and although no intervention studies currently exist, they are considered to have a bidirectional association with neuropsychological outcomes [13]. Caregiver-reported data reveal that children with FASD present with significantly more sleep problems than TD children [14,15]. Caregiver report, polysomnography (PSG), and actigraphy studies report that children with FASD have significantly shorter sleep duration, lower sleep efficiency, more sleep disturbances, and more sleep disordered breathing than TD children [15].

Autism Spectrum Disorders (ASD, hereby autism) are neurodevelopmental conditions characterised by social and communication difficulties, repetitive behaviour, and sensory sensitivities [16]. Decreased working memory in autism appears to be associated with cognitive and behavioural outcomes, such as decreased social communication functioning, alongside increased repetitive behaviours [17], learning paradigms [18], behavioural regulation, and executive control [19]. FMRI studies, such as those conducted by Yeung and colleagues [20], show that when *n*-back tasks were administered to adolescents with autism, compensatory mechanisms can be employed in the right-lateralised prefrontal areas, not used by controls. This suggests that individuals with autism may be employing a different visuospatial processing style or strategies. Working memory in autism tends to be more compromised when serial recall carries specific meaning (such as lexical and semantic meaning) which may be difficult for an individual with autism to decipher. In some domains, individuals with autism may be compensating and recalling at the same rate as typical individuals, whilst others consistently score lower than typical controls [21,22]. Children with autism can show diminished attentional capacity in some instances [23,24], but enhanced attentional ability in other instances. Studies using attentional search tasks reveal that individuals with autism have an enhanced or superior ability to discriminate, but a diminished ability to generalise [25]. Children with autism tend to process higher perceptual loads in attentional tasks, even when increasing task-irrelevant stimuli, which suggests this population has the ability to process a higher perceptual load [26].

Research suggests that sleep is associated with several cognitive domains in autism; however, this literature is scarce in the FASD population. Al-Backer and colleagues [27], for example, report that actigraphy-measured sleep duration is significantly associated with delayed response time in children aged between 7 and 10 years with a diagnosis of autism (*n* = 18). Meanwhile, caregiver-reported insomnia and parasomnia symptoms can predict diminished working memory [28] and hyperactivity [29] in children. PSG data from an adult population reveal a significant negative correlation between slow-wave sleep (SWS) and learning a sensory motor procedural memory task [30]. In addition to cognitive domains, social adaptive difficulties [31], affect [7], repetitive behaviours [32], and communication difficulties [33] are also associated with sleep in the autism population, in toddlers and pre-schoolers as well as adolescents and adults [34]. In the paediatric autism population, sleep disturbances can be improved through behavioural, melatonin, and social story interventions [35,36]; such interventions can impact social interaction, auditory sensitivity, focus, and repetitive behaviour [37]. However, similar causal and correlational data are scarce in the FASD population. Only two published studies have measured the association between sleep and daytime functioning within this clinical population. Sleep disturbances were found to be significant predictors of anxiety in (*n =* 92) children and adolescents with FASD aged between 6 and 16 years [38] and sensory seeking behaviours were significantly correlated in a group of (*n* = 19) children with FASD aged between 3 and 6 years [39]. 

In the present study, a battery of cognitive tasks was used to objectively characterise the different dimensions of attention, working memory, receptive vocabulary, and nonverbal MA alongside objective measurements of sleep parameters. Variables that were controlled for were age, socioeconomic status (SES), and sex, since these are known predictors of cognitive outcomes. The purpose of this exploratory study was to examine the association between sleep and cognitive outcomes in FASD and autism in comparison to a TD control group. It was conducted with the intention of understanding the extent that sleep might explain shared and unique cognitive profiles in these groups.

## 2. Materials and Methods

### 2.1. Participants

**Figure 1:**
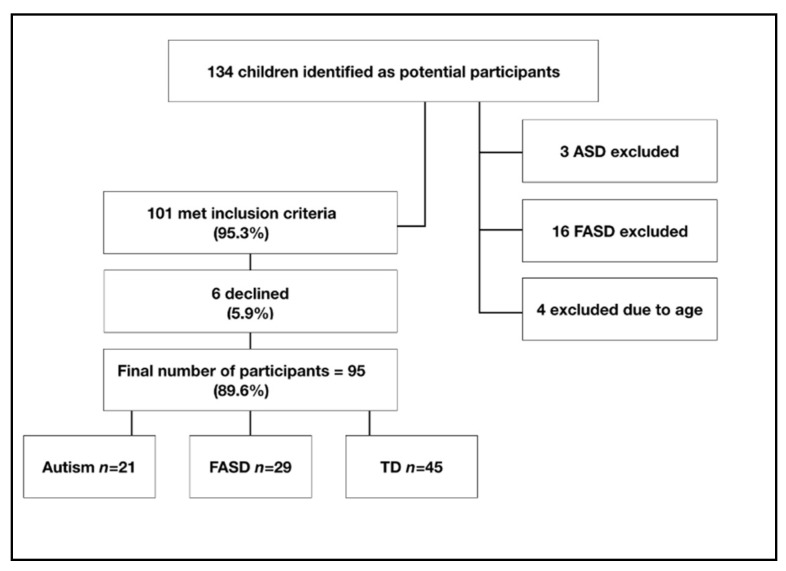
Flow chart detailing number of participants.

Children with a diagnosis of FASD or autism were compared to a TD control group. Details of the child’s diagnosis were provided by the caregiver, including the date, hospital/clinic, and diagnosing professional. All children were aged between 6 and 12 years. Screening tools for both FASD and autism (respectively, Neurobehavioural Screening Tool and Childhood Autism Rating Scale—Parents Version) were administered to ascertain children met diagnostic thresholds. Children with a diagnosis other than FASD or autism were excluded. TD participants were recruited through schools in London. FASD participants were recruited through UK FASD charities, while children with autism were recruited through online autism forums for caregivers. To avoid sample bias, this study was not explicitly advertised as a study of sleep; rather, it was advertised in different ways as a study on cognition, school and learning, home life, social and emotional behaviour, and sleep. One-hundred and thirty-four caregivers responded to the original study advertisements. Of these, 19 were excluded as they did not meet the diagnostic criteria (16 were children with prenatal alcohol exposure who did not have a FASD diagnosis from a clinical professional, 3 were children with autism who had co-occurring diagnoses of ADHD). A further 4 were excluded as they did not meet the age criteria. Out of the remaining 101 families interested in taking part, 95 responded to further communication, signed consent forms, and arranged to take part in the sleep and cognitive testing. This can be viewed in Figure 1. 

### 2.2. Ethical Approval

This study was approved by the UCL Institute of Education Research Ethics Committee (Approval number 16683/001). All caregivers were provided with details of the study, and details on what will happen with the information they provide. Consent was gained from all caregivers, and assent gained from all children where this was understood.

### 2.3. Materials 

#### 2.3.1. Background Questionnaires 

The Childhood Autism Rating Scale, Parents Version (CARS) [40]. This is a 15-item screening questionnaire that determines the severity of autism symptoms, using a seven-point Likert Scale, ranging from typical to atypical behaviour. Categories are: relating to people, imitation, emotional responsiveness, body use, object use, adaptation to change, visual responses, listening responses, taste, smell, touch responses, fear or nervousness, verbal communication, nonverbal communication, activity levels, intellectual responsiveness, and general observations. The CARS demonstrates moderate to good sensitivity and specificity (81.4% and 78.6%, respectively) and good internal consistency (Cronbach’s Alpha = 0.79); however, it cannot be used in place of a diagnostic assessment. A CARS score of ≥33 indicates possible autism [40].

Neurobehavioural Screening Tool (NST) [41]. This is a ten-item binary checklist that screens for possible FASD in children. Questions examine whether children meet the more common neurobehavioural characteristics of FASD; however, these are not always accurate or representative of all children with FASD. Categories are: acting young, lying and cheating, lacking guilt after misbehaving, difficulty concentrating, impulsivity, hyperactivity, displays of cruelty, stealing at home, and stealing outside of the home. The NST has low sensitivity but high specificity (62% and 100%, respectively) and in the absence of a more accurate measurement tool, is a widely used screening mechanism for FASD in children. Scores above 8, plus confirmed prenatal alcohol exposure, indicate probable FASD [41]. 

Socioeconomic (SES) questionnaire. Caregivers were asked their ethnic origin, educational qualifications and job titles of all adults in the household, the number of parents in the household, and whether the child was in foster care, adoptive care, or under the care of a biological parent or relative. SES was determined along the National Statistics Socio-economic Classification of 1 (higher or lower managerial, administrative or professional occupations and/or higher education), 2 (intermediate occupations and A-Levels or equivalent), or 3 (routine or manual occupations or unemployed, some schooling) [42].

#### 2.3.2. Actigraphy

Children were required to wear CamNTech Actiwatch 8 [43] actigraphs continuously for seven days and nights, measuring contiguous epochs of movement throughout the time they were worn. All actigraphy data were collected during term time, which ensured that sleep data reflected a normal school week. The watches were set to the default “medium” sensitivity level and collected one-minute epochs of data. This is in line with previous research which the methodology of the present study replicates [44,45,46]. Consistent settings were used across groups to ensure valid comparisons. Amounts of time were recorded if the level of the signal produced in response to movement during a 1-min time period was above a 0.01 G threshold. The program uses an algorithm to score each one-minute epoch as sleep or wake based on movement during that minute, as well as the two preceding and two successive minutes. Sleep start and sleep end were marked as the start and end, respectively, of a period of 10 or more minutes of immobility. This study was an exploratory analysis of data; the variables included within the analysis are: bed time (the time at which the child fell asleep, measured by contiguous epochs of sleep), wake time (the time at which the child awoke, measured by contiguous epochs of wake/sleep), assumed sleep time (caregiver reported bed times and wake times), actual sleep time (total time spent in sleep according to epoch-by-epoch category), sleep efficiency (actual sleep time expressed as a percentage of time in bed), sleep latency (time between “lights out” and “fell asleep”), number of mean sleep bouts (average length of each sleep bout), mean night waking duration (duration of contiguous sections categorised as wake during the night), mean activity epochs (total activity score during the night), and fragmentation index (an indication of the degree of fragmentation of the sleep period and can be used as an indication of sleep quality) [43]. For clarity of reading, both subjective and objective parameters were included within our analysis. Three children from the FASD group did not tolerate the watch and declined to wear it at all, and three children did not tolerate the watch for the full seven days. Three children from the autism group did not tolerate the watch at all and one participant lost a watch. The final number of participants who completed a minimum of four nights of weekday actigraphy was: autism (*n* = 17), FASD (*n* = 26), TD (*n* = 45).

#### 2.3.3. Sleep Diary

Caregivers completed a sleep diary recording bedtimes, waking up time, any naps or night wakings, and any unusual occurrences or activity. These bedtimes and wake times are reported in the sleep data as “assumed” times and are used as parameters to support the analysis of the actigraphy data. 

### 2.4. Cognitive Tasks

Raven’s Standard Progressive Matrices (RSPM) [47]. The RSPM is a widely used 60 item non-verbal test which measures two components of nonverbal mental age (MA): the capacity to think clearly and make sense of complex data (eductive ability); and the capacity to store and reproduce information (reproductive ability). The test contains five sections which require participants to identify a missing component in a series of figural patterns. The sections, which progressively increase in difficulty, require increasingly greater skill in encoding and analysing information. The RSPM is often used to assess children’s non-verbal mental age (MA) and is a necessary tool when examining children with neurodevelopmental conditions where chronological age (CA) is incongruent with MA. In a sample of 6529 children, Abdel Khalek et al. [48] reported that the RSPM has internal consistency (0.88–0.93 Cronbach Alpha) and good factorial validity (0.73–0.89) [48]. The task was conducted according to the RSPM Manual [45] with no time limit. 

British Picture Vocabulary Scale 3 (BPVS) [49]. The BPVS was used to examine children’s receptive vocabulary and to calculate verbal MA. It consists of 168 words, divided into 14 sets which increase with difficulty. Each set contains twelve words which are read out to the child and shown alongside four pictures. The child is required to point out the picture which corresponds to the word. Children’s vocabulary ages are calculated from raw scores (ceiling item minus error), which correspond to standardised scores, and percentile ranks. From this, the child’s vocabulary age can be calculated. In a sample of 3278, Dunn et al. [49] reported that the BPVS had criterion validity with the Schonell Vocabulary Test of 0.8 and construct validity of 0.71. The task was conducted according to the BPVS Manual [49] with no time limit.

Digit span test of working memory [50]. The digit span test is a subtest from the Wechsler Memory Scale that provides a measure of short-term memory span. The test involves the administrator reading a sequence of digits aloud, after which the participant is required to immediately recall the sequence of digits. A sequence of two digits is read, then three, then four, and so on up to a sequence of nine digits. The participant’s digit span is the longest number of sequential digits that can accurately be recalled. Participants are required to recall digits both forwards and backwards. In a sample of 55 children, Sung [51] reported high test–retest reliability of the digit span test (0.86). In a larger sample of 2200 children, Canivez et al. [52] conducted exploratory and confirmatory factor analysis on the full Weschler Intelligence Scale for Children (WISC), finding that the coefficient for general intelligence was high (0.89) and the coefficients for group factors (including working memory) were lower, ranging from 0.87 to 0.54. The task was conducted according to the WISC Manual [50] with no time limit.

**Figure 2:**
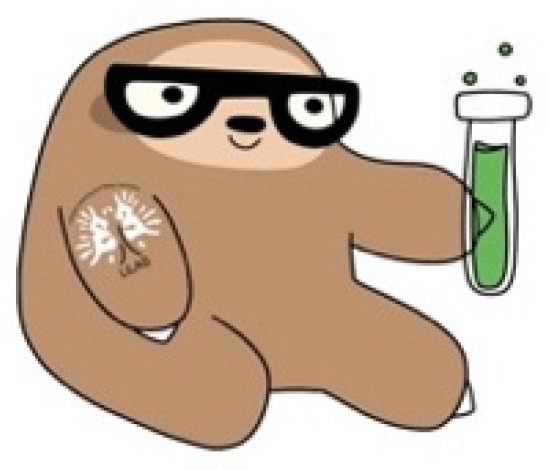
Sloth.

**Figure 3:**
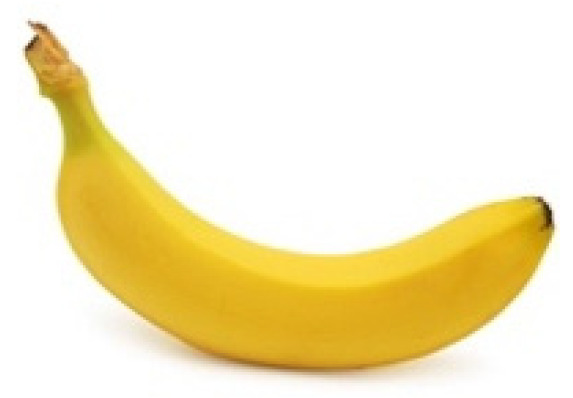
Banana.

Choice reaction time (CRT) continuous performance task [53]. In order to assess children’s sustained attention, vigilance, motor speed, inhibition, and impulsivity, a choice reaction task (CRT) was designed in MATLAB using a PsychTools authorised task [53]. A 2-choice task was used. This is similar to a simple (go–no go) reaction time task, such as the Connors Continuous Performance Test; however, stimulus and response uncertainty are introduced by having two possible stimuli and two possible responses. This is in line with previous CRT tasks that have been used with children with FASD [54] and autism [55]. The task was presented on a MacBook Air laptop with a 33 cm screen and a viewing distance of around 50 cm. The task required the child to respond to two different looking stimuli, a cartoon sloth and a banana, as can be seen in Figure 2 and Figure 3.

When the sloth appeared on the screen, the child was required to press the “left” arrow key. When the banana appeared on the screen, they were required to press the “right” arrow key. Target stimuli appeared on the screen in a random sequence on a white background, with intervals of 0.5–2.00 s. Before the trial, it was ensured that the children were able to identify and discriminate between the objects, and relay the instructions, in order to ensure that the instructions were understood. Children were given verbal instructions: “This is a sloth, and this is a banana. When you see the sloth, you must press this ‘left’ button. When you see the banana, you must press this ’right’ button.” In order to ensure the instructions were understood, further questions were asked: “Which one is the sloth? When he comes up which button do you press? Which one is the banana? When it comes up which button do you press? Brilliant, shall we start?” Correct answers elicited a positive “bell” sound, which is normally associated with correct answers, and incorrect answers elicited a negative “buzz sound”, which is normally associated with negative answers [53]. When necessary, children were given positive feedback and encouraged to continue: “well done, you’re doing great! keep going”. A practice trial, consisting of 10 targets, was administered. The test phase consisted of five blocks of 20 trials. Outcome measures were number of correct and incorrect responses, reaction times for correct and incorrect responses, and impulsivity (measured as percentage of continuous hit reaction times of <100 ms). 

### 2.5. Procedure

All children were requested to wear an actigraph for seven consecutive days and nights. During the week in which children’s sleep was being examined, a battery of tests examining cognitive performance was administered. All tasks were administered in semi-formal test conditions, with background noise at the level of a quiet school classroom. In the clinical groups, caregivers were present either in the room or were nearby, and in the TD group, testing took place in a small, separate room near a busy part of the school. It was ensured that all children understood the tasks before progressing, and assent was acquired after the tasks had been explained by saying: “those are the games and activities that we are going to do today, how does that sound? Would you like to do that, and do you have any questions?”. Children were encouraged to concentrate on the activity but were offered the option of taking a break and returning if needed. 

### 2.6. Statistical Analysis

Data were analysed using the haven, glmnet, and xtable packages in R (R Foundation for Statistical Computing, Vienna, Austria), as well as SPSS 22 for Windows (SPSS Inc. Chicago, IL, USA). Outlying scores were identified through Cook’s distances. Significant results affected by the removal of outliers are indicated here with “OR” (Outlier Removed). 

#### 2.6.1. Group Comparisons

Data were examined for normality using Levene’s Test of homogeneity. To determine whether sex was a confounding factor, independent samples t-tests were used to compare males and females within the FASD and TD groups. Given the uneven ratio of boys to girls in the autism group (17:4), where sex is used as a covariate hereon it does not refer to the autism group. Regressions were used to investigate age-related changes in sleep, nonverbal MA, receptive vocabulary, working memory, and attention. One-way Analysis of Variance (ANOVA) tests were used to determine whether SES differences contributed to either sleep or psychological outcomes, per group. Since some age, SES, and sex differences were found, all subsequent analyses were conducted using age, SES, and sex as covariates. 

Group comparisons between autism, FASD, and TD were made through one-way between-group ANOVAs, for each of the objectively defined variables: sleep data (actigraphy), scores from the RSPM, BPVS, working memory, and attention tasks. In post hoc analysis where equal variances could not be assumed, the Games–Howell test was used. Where equal variances were assumed, the Bonferroni correction was used. Syndrome specificity is defined within this sample as when all three groups were statistically significantly different from each other. 

#### 2.6.2. Regression Analysis

Hierarchical multiple linear regression using the Enter model was used to assess whether sleep was able to predict nonverbal MA, receptive vocabulary, working memory, or attention in autism, FASD, or TD groups. Block one controlled for age, SES, and sex. Tolerance statistics were conducted to examine the collinearity between variables. Actigraphy data were entered into block two. Since there were several highly collinear actigraphy variables (>0.9), separate analyses were conducted for each actigraphy variable (bed time, wake time, assumed sleep time, actual sleep time, sleep efficiency, sleep latency, mean sleep bout duration, number of night wakings, mean night waking duration, mean activity epoch, fragmentation). These variables were analysed separately in order to avoid type II errors, and additionally to conduct an exploratory analysis of the association between sleep and cognition. Adjusted *R*^2^ values are reported as the percentage of variance, in order to control for the number of predictors in the model. Two-one-sided tests were conducted in R to assess for significant similarities between groups for all variables. 

## 3. Results 

### 3.1. Demographic Data

One-way between-group ANOVAs indicated differences in age (F(_1,93_) = 1.06, *p* = 0.03, η_p_^2^ = 0.09), SES (F(_1,3_) = 1.06, *p* = 0.04, η_p_^2^ = 0.08), and sex (F(_1,2_) = 6.58, *p* = 0.01, η_p_^2^ = 0.02). There were significantly more boys than girls in the autism group, but no significant sex differences in the TD and FASD groups. Because of the heterogeneity of samples, all regression analyses were conducted with SES and CA as covariates. Regression analyses in the FASD and TD groups, additionally, contained sex as a covariant, but not the autism group. The final sample consisted of 95 participants, outlined in Table 1.

### 3.2. Sleep Characteristics Based on Actigraphy

**Figure 4:**
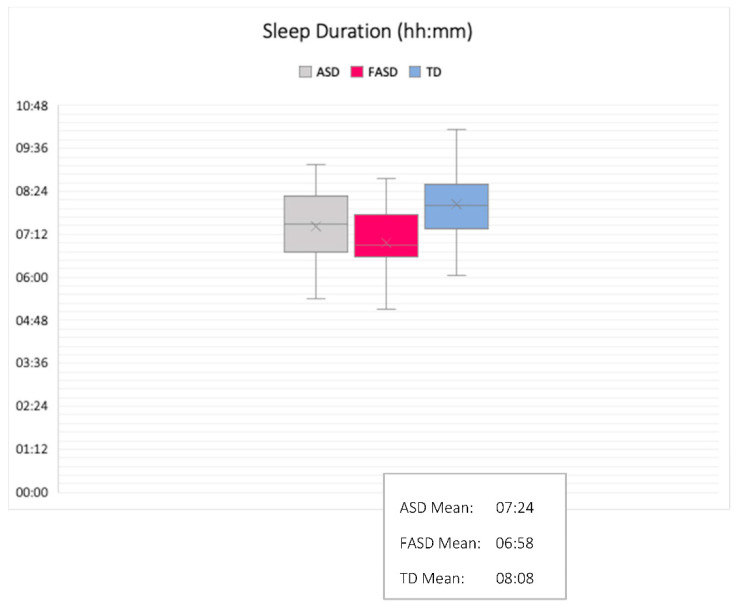
Sleep duration (hh:mm): comparison between groups (M/SD shown).

**Figure 5:**
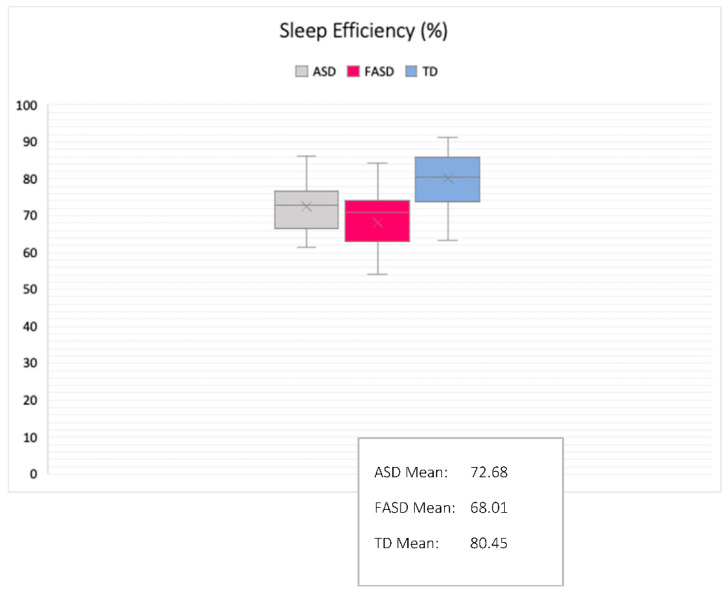
Sleep efficiency as a percentage: comparison between groups (M/SD shown).

**Figure 6:**
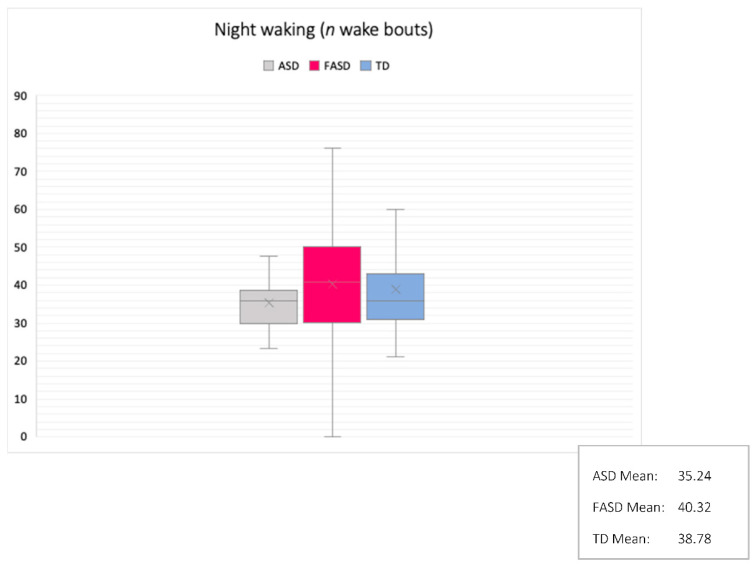
Mean night wakings (n): comparison between groups (M/SD shown).

Group comparisons were made using ANOVA and tests of similarity. Comparisons of group differences revealed significant effects in actual sleep time, sleep efficiency, mean sleep bouts, night waking duration, and fragmentation index. Partial eta squared indicated medium and large effect sizes (all above 0.0588). Group differences and similarities are outlined in Table 2 and Figure 3, Figure 4, Figure 5 and Figure 6.

#### 3.2.1. Chronological Age (CA) and Sleep

Bedtime was significantly related to CA in the TD group, with older children going to bed later. It was not significantly related to CA in the autism or FASD groups (autism: *R*^2^ = 0.09, F_(1,17)_ = 1.43, *p* = 0.25; FASD: *R*^2^ = 0.02, F_(1,25)_ =0.45, *p* = 0.51; TD: *R*^2^ = 0.10, F_(1,39)_ = 3.39, *p* = 0.05). Wake time significantly changed with age for the TD and autism groups. Older TD children had significantly later wake times, but older children with autism had significantly earlier wake times, regardless of the day of the week. There were no age-related changes in the FASD group (autism: *R*^2^ = 0.22, F_(1,15)_ = 3.92, *p* = 0.03; FASD: *R*^2^ = 0.003, F_(1,25)_ = 0.6, *p* = 0.78; TD: *R*^2^= 0.11, F_(1.39)_ = 3.5, *p* = 0.04). 

The mean number of sleep bouts increased significantly with age for children with autism, but not for TD children or children with FASD (autism: *R*^2^ = 0.15, F_(1,15)_ = 3.66, *p* = 0.04; FASD: *R*^2^ = 0.03, F_(1,24)_ = 0.29, *p* = 0.29; TD: *R*^2^ = 0.01, F_(1,39)_ = 1.39, *p* = 0.12).

Some effect sizes were too small to state a significant result, but some β values in the variables of assumed sleep, sleep duration, sleep efficiency, sleep latency, and fragmentation showed either small developmental trends, or no change with increased chronological age. These can be seen in Table 3. 

#### 3.2.2. Socioeconomic Status (SES) and Sleep

SES was not significantly associated with bedtime in the clinical groups, but in the TD group, SES was significantly related to bedtime, with higher SES participants sleeping earlier (F_(1,17)_ = 4.60, *p* = 0.038, *R*^2^ = 0.11).

#### 3.2.3. Sex Differences and Sleep

In the FASD group, girls had significantly later wake times than boys (male: *m* = 06:17, SD = 1:18, female: *m* = 07:22, SD = 00:58; *t*(24) = −2.4, *p* = 0.02). In the TD group, boys moved significantly more at night, with mean activity epochs significantly higher than girls (male: *m* = 27.98, SD = 9.97, female: *m* = 21.32, SD = 10.87; *t*(39) = 2.04, *p* = 0.04).

### 3.3. RSPM/Non-Verbal MA

The test was completed by all TD participants, 20/21 autism, and 24/29 FASD participants. Non-verbal MA is calculated using the RSPM total score, out of a possible 36. Two children (one with autism, one FASD) scored below the threshold of 5 for calculating MA; thus, all analyses herein use RSPM total score, as described by Ashworth [44].

There were significant differences between the autism, FASD, and TD groups in the RSPM total scores, indicating distinct clinical profiles, significantly different to TD populations (see Table 4). There were no significant associations between the RSPM scores, SES, and sex. CA was significantly associated with RSPM scores in all three groups (autism: *R*^2^= 0.22, F_(1,19)_ = 5.26, *p* = 0.03. FASD: *R*^2^ = 0.50, F_(1,23)_ = 22.15, *p* ≤ 0.001. TD: *R*^2^= 0.57, F_(1,44)_ = 55.81, *p* ≤ 0.001). 

### 3.4. BPVS/Receptive Vocabulary

The test was completed by all autism and TD participants, and 25/29 FASD participants. There were significant differences between autism and FASD, and TD and FASD groups in the BPVS standard scores; however, there was no significant difference between autism and TD groups (see Table 4). Children in the FASD group consistently scored lower than the other two groups. 

There were no significant associations between BPVS scores and sex in the FASD and TD groups. There were significant associations between BPVS scores and SES in the autism and TD group, with higher SES groups performing better on the task (autism: ß = 0.39; *p* = 0.05. FASD: ß = 0.23; *p* = 0.07. TD: ß = 0.33; *p* = 0.041).

Higher CA was associated with a higher BPVS score (autism: *R*^2^ = 0.21, F_(1,20)_ = 5.21, *p* = 0.03. FASD: *R*^2^ = 0.46, F_(1,24)_ = 19.24, *p* ≤ 0.001. TD: *R*^2^= 0.04, F_(1,44)_ = 1.64, *p* = 0.01). 

### 3.5. Digit Span Test of Working Memory

The test was completed by all autism and TD participants and 24/29 FASD participants. Outcome measures were noted as raw score forwards and raw score backwards. Regression models accounted for age. One-way ANOVA results and two-one-sided tests of similarity showed that there were significant differences and similarities between digit span forward and backward raw scores among the groups (see Table 4).

CA was significantly related to forwards and backwards raw scores in the TD and FASD groups, but not in the autism group, with older children tending to achieve higher results (Forwards: autism: *R*^2^ = 0.24, F_(1,21)_ = 5.99, *p* = 0.02. FASD: *R*^2^ = 0.45, F_(1,23)_ = 18.25, *p* ≤ 0.001. TD: *R*^2^ = 0.26, F_(1,44)_ = 15.36, *p* ≤ 0.001; Backwards: autism: *R*^2^ = 0.16, F_(1,20)_ = 3.54, *p* = 0.08. FASD: *R*^2^ = 0.33, F_(1,23)_ = 10.29, *p* = 0.03. TD: *R*^2^ = 0.45, F_(1,44)_ = 34.54, *p* ≤ 0.001). SES and sex were not associated with digit span results in any of the groups. 

### 3.6. Attention

The task was completed by all autism and TD participants, and 22/29 FASD participants. One-way ANOVA results showed that there were significant differences in correct responses, incorrect responses, reaction times, and impulsivity. There were no significant differences or similarities between the autism and TD groups in commissions, or in omissions or impulsivity (see Table 4). 

There were significant sex differences in correct responses in the FASD group, with boys showing lower levels of vigilance than girls (male: M = 78.92, SD = 29.38, female: M = 94.78, SD = 3.99. t(20) = 1.59, *p* = 0.03). There were no significant associations between sex or SES. CA was significantly related to reaction time in the TD, but not in the clinical groups. Older children had higher correct reaction times (autism: *R*^2^= 0.12, F_(1,20)_ = 9.05, *p* = 0.12. FASD: *R*^2^ = 0.08, F_(1,22)_ = 2.67, *p* = 0.19. TD: *R*^2^= 0.31 F_(1,44)_ = 10.67, *p* ≤ 0.001).

### 3.7. Regressions between Sleep and Cognition

Significant regressions between sleep, nonverbal MA, receptive vocabulary, working memory, and attention were found in the autism, FASD, and TD groups. Within the FASD group, several significant associations were found between the RSPM and actigraphy parameters, as well as the BPVS and actigraphy parameters. Within the TD and autism groups, several actigraphy parameters were significantly associated with attention and working memory (see Table 5). 

## 4. Discussion

Although sleep problems and their daytime effects are often reported by caregivers of children with FASD and autism [15,29], the scientific literature on this topic remains scarce. In the present study, we measured the sleep and cognitive profiles of children with FASD and autism with the intention of comparing these two clinical populations. All actigraphy variables were analysed in this exploratory study. We found “syndrome specificity” (which we describe as significantly different scores between each group) within the sleep profiles, as well as significantly more disrupted and shorter sleep within the clinical samples than the TD sample. We report that children in the clinical groups presented with differing cognitive profiles, and within the TD and autism samples, higher cognitive scores were positively correlated with the quantity and quality of sleep. Within the FASD sample, however, a differing pattern of correlations was found, which shall be addressed below.

### 4.1. Sleep Differences between Groups

Sleep duration, sleep quality, sleep bouts, night wakings, and fragmentation were significantly different between the three groups, suggesting syndrome specificity. Children with FASD slept for an average of 6 h and 58 min, with an average of 68% sleep efficiency and a fragmentation score of 40. Children with autism slept for an average of 7 h and 24 min, with an average of 72% sleep efficiency and a fragmentation score of 36, whilst TD children slept for an average of 8 h and 6 min, with an average of 80% sleep efficiency and a fragmentation score of 31. Meanwhile, tests of significant similarity revealed that bedtimes, wake times, and assumed sleep were similar in the autism and FASD groups. This means that even though children with autism and FASD went to sleep at similar times, had similar wake times, and caregivers thought they slept statistically similar amounts, children with FASD had poorer sleep than children with autism within the time in which they were asleep. This implies that night waking should be higher in the FASD group than the autism group: children with FASD had increased fragmentation compared to both the autism and TD groups which may explain why, despite the fact that bedtimes and wake times were statistically similar between the two clinical groups, sleep efficiency and duration was poorer in the FASD group. Thus, several sleep parameters appeared to be syndrome-specific. Amongst other things, this specificity suggests that the neural mechanisms of sleep may mature and develop differently depending on the structural and functional differences that make up autism and FASD. There are multiple reasons that sleep quality may be impaired in the two clinical groups (for example, factors relating to environment, health, sleep disorders as well as underlying brain structure), but one suggestion is that the cortical and subcortical maturation that is associated with NREM (and some REM sleep-dependent neural maturation) may have a different developmental trajectory in autism and FASD. Further evaluation using multidisciplinary methodologies would be beneficial here. 

Sleep was more disrupted and shorter in the clinical groups than the TD group. Our results are in contrast to Wengel and colleagues [39], who report that although sleep onset latency was significantly longer than age-matched controls in their clinical group of 3–6-year-old children with FASD (*n* = 19), actigraphy-measured sleep quality and duration did not differ. The inconsistency in results between the present study and Wengel et al. [39] may be due to the differences in the younger age of participants in the Wengel et al. study, differences in sample sizes, and/or differences in the types of actigraph used. Children with FASD can reach age-appropriate developmental milestones in the early years, but social, emotional, and cognitive development tends to delay at the start of school and plateau at around seven years old; hence, the minimum age that a child can be referred to a FASD clinic is six years, at the time of the developmental plateau [9,10]. The present results suggest that if 3–6-year-old children do not experience significant sleep disturbances, but 6–12-year-old children do, perhaps sleep disruption also follows a similar developmental trend, plateauing (or perhaps regressing) at the same time as the cognitive domains. 

The present study findings are somewhat consistent with previous studies assessing sleep in children with autism; however, previous findings are not easily generalisable. A meta-analysis by Díaz-Román and colleagues [56] reported on five paediatric studies that met their quality criteria for actigraphy findings in children with autism. Their parameters were: sleep onset latency, actual sleep time, assumed sleep time, actual wake time, and sleep efficiency. In this meta-analysis, children with autism (*n* = 140) slept on average 7 h and 38 min, with an average of 73% sleep efficiency, whilst TD children (*n* = 132) slept on average 8 h, with an average of 90% sleep efficiency. Sleep was also more fragmented in the autism than TD group, and standard mean differences with 95% CI showed that children with autism consistently presented with more sleep problems than TD children. However, within the same sample, sleep efficiency, sleep duration, fragmentation, and assumed sleep were heterogenous, which indicates the difficulty of generalising these kinds of findings. Inconsistent with previous studies, our sleep onset latency results were not significantly higher in the autism and FASD group than the TD group; however, sleep onset latency data can be unreliable, particularly when it can depend on parental report. We also found no significant differences in children’s bedtimes, wake times, or assumed sleep; however, some nonsignificant trends were found. 

### 4.2. Cognitive Profiles

Nonverbal MA scores were significantly different between all three groups, which we define as syndrome specificity within our sample. There were some differences between the FASD and autism group in the assessment of the BPVS, but no differences between the autism and TD groups. We found that TD children had the highest mean MA, and the FASD group had the lowest, which was expected given the higher levels of learning difficulties in children with FASD [10]. Both clinical groups also experienced additional environmental pressures such as performance anxiety and cognitive exhaustion, which can have an impact on cognitive scores. In all three groups, MA and CA were linearly related. In addition, previous work on FASD populations outlines the impact of the disruption caused by change in caregiver [57]. In the present study, 28 out of 29 children with FASD were in the care of an adult other than the birth parent. Whilst it is beyond the scope of this study to speculate on the cognitive and behavioural impact of foster care on the atypical developmental trajectory of the child, such extraneous variables are important in FASD research.

In the attention task, children with FASD had fewer correct responses and were slower to react to the choice stimulus than children in the other two groups. In children with autism, delays in choice reaction have been noted as the result of an intact ability to execute a movement but delayed ability to prepare for it [58], which can account for the non-significant results between the TD and autism groups in correct and incorrect responses, but the difference in results for reaction times. Previous work on sustained attention in children with prenatal alcohol exposure [55] shows that this population tends to have higher levels of inattention and lower task performance than TD children, which is supported by the present data.

Due to cortical damage as a result of prenatal alcohol exposure, working memory, short term memory, and memory consolidation problems are among the main cognitive issues in children with FASD [9]. Tests of working memory in the autism population are more heterogeneous [58], relying on visuospatial, phonological, attentional, and executive control domains [21,58]. The present findings established working memory differences between the clinical and TD groups, whilst significant similarities emerged between the autism and FASD groups on logarithmic digit span backward scores. If this is examined within the working memory model of Baddeley and Hitch [59], it suggests there might also be similarly impaired visuospatial, phonological, attentional, and executive control functions in the two conditions. However, in the present study and in previous ones, sustained attention tends to be significantly different between the two groups. It may be the case that methodological issues in the present study have not offered a full or substantial picture of the working memory model, but if replicated studies obtain similar results, perhaps the working memory model is not applicable to these clinical groups and thus, suggests syndrome-specific differences in attentional and executive control domains. It is beyond the scope of this paper to make an argument for neuroconstructivism, but further studies in this field should assess prenatal alcohol exposure-affected cortical and subcortical structural damage (evidenced in FASD), in comparison with overcompensated localised neural connectivity (evidenced in autism). Both divergent neural pathways result in significantly similar “impaired” domains, as well as advanced “intact” ones such as the superior attentional abilities as mentioned above [60]. 

### 4.3. Sleep and Cognition

The regression models used here demonstrate that several sleep parameters are associated with cognitive outcomes in the TD, autism, and FASD groups, but some results were inconsistent with the previous meta-analytical literature. In the TD group, sleep parameters accounted for 26–32% of the variance in working memory and 24% of the variance in correct hit rate, with longer sleep duration and higher sleep efficiency accounting for higher cognitive scores. In the autism group, actual sleep accounted for 24% of the variance in working memory scores, with longer sleep duration accounting for higher working memory scores. Sleep fragmentation accounted for 31% of the variance in impulsivity, with higher fragmentation scores indicating a higher impulsivity rate. Likewise, longer sleep duration was associated with higher rates of correct hits in the attention task. In the FASD group, however, it appeared that higher scores in the cognitive tasks were associated with higher rates of sleep disturbances: more mean night wakings, higher fragmentation, lower sleep efficiency, and later bedtimes were associated with higher cognitive scores. 

TD children who experienced higher sleep efficiency performed better in the digit span test. This is consistent with previous work on the association between sleep and working memory in neurotypical individuals, where in paediatric populations, sleep disturbance is associated with reduced performance in working memory tasks [2]. This performance decline is mediated by neural connectivity in the frontal and parietal areas [61]. Since working memory is an important part of cognitive performance, reduced ability in this domain may be associated with reduced task performance in several neurocognitive domains. 

Additionally, children with autism who slept longer performed better on the test of working memory. This is supported by work by Calhoun and colleagues [22] where, in a sample of adolescents with autism (*n* = 96), digit span tests and actigraphy revealed that poorer working memory was linearly related to increased sleep disturbances. In the FASD sample, however, the only sleep parameter to be associated with working memory was bedtime: the later the child’s bedtime, the longer the digit span. One obvious reason for this is that older children had later bedtimes and therefore, longer digit spans were related more to the developmental trajectory, despite CA being controlled for in the model. 

In summary, sleep duration was a significant predictor of working memory and attention in the TD and autism groups, but not in the FASD group. Elsewhere, strong associations have been found between sleep and anxiety, and sleep and sensory seeking behavioural issues in children with FASD [38,39]. This indicates that there may be a difference between psychological domains of affect, behaviour, and cognition in this population, and their susceptibility to sleep. Other than bedtime, neither working memory nor attention appeared to be significantly associated with sleep in the FASD group (although nonsignificant results with smaller effect sizes did show associations between sleep and cognition). It is unclear why these relationships are inconsistent between the groups, but one reason may be due to the underlying structural damage to the prefrontal areas caused by prenatal alcohol exposure. Astley and colleagues [12] conducted fMRI assessments on a sample (*n* = 58) of children with FASD, whilst administering the *N*-back working memory task, in which amongst a series of faces that were presented, participants were required to identify duplicate consecutive and non-consecutive images. Performance was poorer in the FASD sample than control sample, and performance on the task was marked by significant deficits in long-range prefrontal, posterior, and parietal lobe function. Hence, working memory problems are intrinsic to the FASD neurocognitive profile due to this functional deficit [9]. Meanwhile, in TD populations, prefrontal and parietal areas continue to mature into adolescence and are thought to have an association with sleep, since this later maturation makes these areas vulnerable to the effects of sleep disruption [62]. Firstly, one of the functional processes of slow-wave sleep is the consolidation of memory through global cortical and subcortical areas. During this time, prefrontal areas appear to “functionally disconnect” from other regions [61]. Secondly, after sleep deprivation has occurred, prefrontal areas are less able to attend to cognitively demanding tasks, and theta waves can be observed which correspond with diminished working memory function and sustained attention [1]. It may be the case that in FASD, global structural and functional damage due to prenatal alcohol exposure has caused working memory deficits, which, since they are intrinsic to FASD, will not be associated with sleep to a significant extent. Or perhaps sleep and cognition are part of a reciprocal association, rather than a linear causal one. It is unclear whether this association would improve with sleep intervention, but this result demonstrates the need for this area of research.

## 5. Conclusions

This is the first study to examine sleep and its association with cognition in individuals with FASD, and to compare sleep in individuals with FASD and autism. Sleep was significantly associated with several cognitive domains in all three groups, and sleep disturbances were observed at significantly higher rates in the clinical groups than the TD group. Our findings support previous studies suggesting that sleep is a clinical concern for FASD and autism populations. It is also proposed here that within these two clinical populations, there exists a complex interplay between sleep and several cognitive domains that are crucial during development. 

Whilst controlled settings and standardised tasks measure cognitive ability, they also require that the child is not experiencing sensory, perceptual, or cognitive overload. The present study, and many previous large-scale ones, have reported the diminished intellectual abilities of children with FASD through measuring ability on standardised scales (e.g., [63,64,65]). These studies have noted that children with FASD consistently score lower on tests of cognitive ability but one limitation here is that cognitive exhaustion, performance anxiety, and negative school experiences may cause children to withdraw from laboratory or classroom-like settings. 

Children with autism and FASD can show advanced sustained attention when attending to particular games, videos, or interactive activity, but may not show the same motivational attention when presented with certain cognitive attention tasks. This may mean that the child obtains a low score on a controlled attentional task but is actually capable of longer sustained attention [64]. Conversely, a ceiling effect emerged within the attention task in the TD group, with 16 children (36%) making no errors. We attempted to mitigate these factors by creating minimal perceptual overload and a relaxed environment for children. 

Actigraphy was used to assess sleep over a period of time within the child’s home setting. Actigraphy is particularly useful for research with populations with sensory issues and sensitivity to change or laboratory settings; however, it is widely recognised that PSG is the gold standard of sleep research. 

Finally, whilst attempts were made to refrain from emphasising this as a sleep study, it was explicit that the characteristics of children’s sleep would be measured. For this reason, there may be a sample bias within the clinical groups as caregivers with children with sleep problems were more likely to take part. 

Subsequent work could see whether modifying sleep can change these cognitive outcomes. Sleep assessments and interventions should be designed specifically for children with autism and FASD, given the variability of their neurodevelopmental profiles as well as their apparent sensitivity to sleep disturbances.

Future studies will test the robustness of the claim that there is an association between daytime functioning and sleep in these clinical populations. In turn, it must be ascertained whether this correlational association is causal. Sleep intervention studies are much needed both in FASD as well as autism and are areas of immediate concern, since interventions may improve cognition, behaviour, and affect. Additionally, the underlying neuropsychological mechanisms would be better understood through neuroimaging studies. Sleep problems are a burden on caregivers, family, and the children involved. Sleep intervention and correlative studies can contribute to the understanding of the function and impact of sleep in autism and FASD, with the ultimate aim of alleviating some of the stresses and burdens faced by children with autism and FASD, as well as their caregivers. 

## Figures and Tables

**Table 1:** Participants.

	Autism (*n* = 21)	FASD (*n* = 29)	TD (*n* = 45)
Male/Female	17/4 ^1^	16/13	23/22
Age (M/SD)	8.42(1.81)	9.60(2.48) ^2^	8.12(1.29)
SES 1/2/3	5/15/1	1/20/8	6/29/10
Living with Biological parent	21	1 ^3^	44
Living with Foster parent	0	22	1
Living with Adoptive parent	0	4	0
Living with Biological relative	0	2	0
Co-occurrence	-	SPD (*n* = 2); ADHD (*n* = 2)	-

^1^ Significant differences between ASD/FASD and ASD/TD. ^2^ Significant difference between FASD/ASD. ^3^ Significant differences between FASD/ASD and FASD/TD (significant differences calculated as <0.05). ASD—autism spectrum disorders; FASD—fetal alcohol spectrum disorders; TD—typically developing.

**Table 2:** Group differences for actigraphy.

	Autism (*n* = 17)		FASD (*n* = 26)		TD (*n* = 45)				
	Mean	SD.	Mean	SD.	Mean	SD.	F	*p*	np^2^
Bed Time	21:18:45	0:57:26	21:14:50	1:21:32	21:08:13	0:55:32	0.28	0.75 ^1^	0.01
Wake Time (hh:mm:ss)	0:07:03	0:01:13	0:07:06	0:01:10	0:06:59	0:00:43	0.13	0.88 ^1^	0.00
Assumed Sleep Time (hh:mm:ss)	9:12:52	1:00:52	9:29:20	0:57:11	9:45:58	1:18:25	1.42	0.25 ^1^	0.03
Actual Sleep Time (hh:mm:ss)	7:24:33	1:03:03	6:58:41	1:11:07	8:06:55	1:04:44	8.74	**<0.001** ^2,3,4^	**0.18** *
Sleep Efficiency (%)	72.68	7.55	68.00	10.82	80.02	6.99	16.39	**<0.001** ^2,3,4^	**0.29** *
Sleep Latency (hh:mm:ss)	0:38:18	0:34:12	0:24:30	0:20:20	0:26:08	0:26:49	1.53	0.22	0.04
Mean Sleep Bout (hh:mm:ss) Night Wakings (*n*)	0:24:21 35.24	0:33:54 7.20	0:11:00 40.32	0:03:21 16.73	0:13:50 38.61	0:04:07 13.65	4.10 0.67	**0.02**^2,3^ 0.51	**0.14** * 0.02
Mean Night Waking (hh:mm:ss)	0:03:07	0:00:46	0:04:18	0:03:31	0:02:36	0:01:03	4.99	**0.01** ^2,3^	**0.11** *
Mean Mobile Activity	18.15	8.24	30.32	32.47	24.73	10.83	1.85	0.16	**0.09** *
Fragmentation Index	31.06	7.60	40.44	17.08	36.01	9.76	2.96	**0.05** ^2,3,4^	**0.07** *

^1^ Significant similarity between autism and FASD (*p* ≤ 0.05); ^2^ Significant difference between autism and TD (*p* ≤ 0.05); ^3^ Significant difference between FASD and TD (*p* ≤ 0.05); ^4^ Significant difference between autism and FASD (*p* ≤ 0.05). * Medium effect size. Significant results shown in bold.

**Table 3:** Nonsignificant results.

	β	*R* ^2^	*p*
*Assumed Sleep/CA*			
Autism	**0.26**	0.07	0.79
FASD	−0.15	0.02	0.10
TD	**0**	<0.01	0.72
*Sleep Duration/CA*			
Autism	**−0.33**	0.11	0.14
FASD	−0.14	0.02	0.49
TD	**−0.02**	<0.01	0.91
*Sleep Efficiency/CA*			
Autism	0.11	0.01	0.69
FASD	−0.16	0.03	0.43
TD	**0.02**	<0.01	0.89
*Sleep Latency/CA*			
Autism	**−0.22**	0.05	0.42
FASD	0.07	0.01	0.72
TD	**0.01**	<0.01	0.93
*Fragmentation/CA*			
Autism	−0.07	0.01	0.79
FASD	**0.21**	0.04	0.31
TD	0.06	<0.01	0.72

Highlighted results: β absolute value of >0.2 indicates a slight but not significant trend, whilst < 0.05 indicates a constant or no developmental trend. CA—chronological age. Significant results shown in bold.

**Table 4:** Between-groups one-way ANOVA for RSPM, BPVS, CRT, and Digit Span.

	Autism (*M (SD))*	FASD (*M (SD))*	TD (*M (SD))*	*f*	Sig	Autism/ TD	FASD/ TD	Autism/ FASD
RSPM Score	22.38	18.75	26.82	2.09	0.04	0.04	0.001	0.003
	(8.81)	(10.01)	(6.83)					
BPVS Standard Score	95.09	87.16	98.91	5.136	**0.01**	0.79 *OR*	0.01 *OR*	0.05
	(15.79)	(15.12)	(13.95)					
Correct Responses (*n*)	86.47 (9.75)	85.41 (23.72)	93.8 (7.79)	3.56	**0.03**	0.12	**<0.001**	**0.05**
Reaction Time Correct (*ms*)	1162.17 (776.78)	942.27 (370.74)	862.86 (289.58)	2.93	**0.05**	**0.04**	**0.05**	**0.05**
Reaction Time Incorrect (*ms*)	799.67 (461.76)	2577.27 (8435.94)	683.61 (405.93)	3.56	**0.03**	**0.01**	**0.05**	**0.02**
Impulsivity	4.14 (6.69)	5.36 (21.23)	0.27 (1.27)	3.88	**0.01**	**0.03**	**0.04**	0.62
Digit Span Forward	20.62 (7.02)	17.04 (6.33)	22.73 (5.30)	7.02	**0.001**	0.21	**0.001**	**0.03**
Digit Span Backward	10.1 (5.30)	9.92 (4.37)	13.47 (5.86)	4.12	**0.02**	**0.05**	**0.02**	0.43 *

* Significant similarity between autism and FASD (*p* ≤ 0.05). RSPM—Raven’s Standard Progressive Matrices; BPVS—British Picture Vocabulary Scale 3; CRT—choice reaction time. Significant results shown in bold.

**Table 5:** Significant regressions between cognition and sleep.

	Autism (*n* = 20)					FASD (*n =* 25)						TD (*n* = 45)			
	B	SEB	ß	*R* ^2^	*p*	B	SEB	ß	*R* ^2^	*p*	B	SEB	ß	*R* ^2^	*p*
*RSPM*															
Bedtime	<0.001	0	0.11	0.37	0.66	<0.001	<0.001	0.32	0.48	**0.01**	0.01	0	0.32	0.6	**0.01**
Sleep Efficiency	0.1	0.07	0.35	0.12	0.18	−0.1	0.06	−0.5	0.18	**0.02**	−0.1	0.1	−0.1	0.02	0.57
Wake time	0.09	16.5	0	<0.001	0.99	76.1	23.8	0.58	0.34	**0.004**	44.8	80.6	0.09	<0.001	0.59
Mean night waking	−0.3	4.94	−0.2	0.03	0.56	2.89	1.4	0.42	0.14	**0.05**	−0.2	0.95	−0.3	0.03	0.87
Mean mobile activity	0.01	0.09	0.02	<0.001	0.96	0.42	0.14	0.56	0.28	**0.01**	−0.1	0.16	−0.1	0.01	0.65
*BPVS*															
Bedtime	<0.001	0	0.11	0.37	0.66	<0.001	<0.001	0.32	0.48	**0.01**	0.01	0	0.32	0.6	**0.01**
Sleep Efficiency	1.78	1.28	0.34	0.16	0.18	−2.1	0.86	−0.5	0.22	**0.03**	−0.4	0.35	−0.2	0.04	0.23
Mean mobile activity	−0.1	1.13	−0.1	0.01	0.96	1.05	0.31	0.59	0.36	**0.003**	−0.1	0.23	−0.1	0.09	0.55
Sleep Fragmentation	−1.6	1.26	−0.3	0.1	0.23	0.97	0.41	0.44	0.21	**0.03**	−0.5	0.26	−0.3	0.01	0.84
*Reaction Time Correct*															
Sleep Bouts	1.25	0.31	0.29	0.32	**0.04**	0.97	0.4	0.09	0.03	0.57	2.97	0.34	0.47	0.24	**0.01**
*Impulsivity*															
Sleep Fragmentation	0.85	0.03	0.39	0.31	**0.01**	1.23	1.11	0.06	0.01	0.32	2.32	1.24	0.12	0.14	0.34
*Digit Span Forwards*															
Sleep Efficiency	1.78	0.01	0.33	0.24	**0.01**	0.02	0	0.19	0.09	0.21	1.68	0.01	0.41	0.32	**0.01**
*Digit Span Backwards*															
Bedtime	1.68	0.12	0.42	0.21	0.22	2.36	0.21	0.54	0.29	**0.05**	1.7	0.02	0.38	0.26	**0.04**
Actual Sleep	1.35	0.15	0.39	0.24	**0.01**	2.55	1.01	0.03	0.42	0.23	0.01	0	0.01	0.05	0.57

Significant regressions shown in bold.
